# Application of a Nanoporous Metal Organic Framework Based on Iron Carboxylate as Drug Delivery System

**Published:** 2018

**Authors:** Bahare Miri, Negar Motakef-Kazemi, Seyed Abbas Shojaosadati, Ali Morsali

**Affiliations:** a *Biotechnology group, Chemical Engineering Faculty, Tarbiat Modares University, Tehran, Iran.*; b *Department of Medical Nanotechnology, Faculty of Advanced Sciences & Technology, Pharmaceutical Sciences Branch, Islamic Azad University, Tehran, Iran (IAUPS).*; c *Department of Chemistry, Faculty of Sciences, Tarbiat Modares University, Tehran, Iran.*

**Keywords:** Metal Organic Framework, MIL-101-NH_2_-Fe, 5-flurouracil, drug delivery system

## Abstract

In the present study, a nanoporous metal organic framework (MOF) based on iron metal and amino terephthalate ligand MIL-101-NH_2_-Fe has been used as a carrier for loading and *in-vitro* release of 5-flurouracil (5-FU) anticancer drug. The 5-FU drug loaded MOF was 13 wt % by using thermogravimetric analysis (TGA). The 5-FU release was monitored under physiological condition at 37 °C, pH 7.4 in simulated body fluid (SBF) by using spectrophotometry. The drug demonstrated a slow release profile where 98% of the drug was released in 4 days. Loading of drug was characterized by Fournier transform infrared (FTI-IR) and thermogravimetric analysis (TGA). The crystalline structure was monitored by using X-ray powder diffraction (XRD) and after loading of drug in the MOF, the pattern of samples was remained the same. The morphology and size of samples were showed by using scanning electron microscopy (SEM) and based on the MOF has a length of 500 nm and an average diameter of 200 nm. These structural characterizations were performed to verify the 5-FU drug loading in MIL-101-NH_2_-Fe. The MOF stability was studied by measuring the iron concentration in the SBF solution with atomic absorption spectroscopy (AAS). The MTT assay method was assessed the ability of this drug delivery system on overcoming MCF-7 breast cancer cells in comparison with the free drug and the carrier alone. Based on the results, this drug loaded nanoparticle could achieve more cell death as compared to the free 5-FU drug.

## Introduction

Drug delivery is becoming a broad area of research, with a variety of activities such as, targeted drug delivery to the proper site of action, control of drug release kinetics, and design of drug formulations (1). Controlled release technology in drug delivery has been used and continued to expand rapidly since 1970s. Among the various existing drug delivery systems, porous materials are emerging as a new class of host/guest systems (2). Currently porous coordination polymers (PCPs) have received considerable attentions because of their unique properties (3). PCPs, also called Metal-organic frameworks (MOFs). These structures have grafted from metal connecting points and organic bridging ligands (3, 4). In recent years, MOFs wildly have been studied as absorbent for molecule separation (5), catalyst (6), optical (7), luminescent (8), magnetic (9) and electronic (10). Recently, the potential application of these materials in biomedical fields such as drug delivery attracted the attention (11). Among the tens of thousands of known MOFs, the MIL family has built from trivalent metal centers and dicarboxylate bridging ligands attracted attention because of their enhanced stability, enormous porosity, and very large pores (12). 

The 5-FU drug is anticancer drug for the treatment of breast cancer. 5-FU is a thymidylate synthase inhibitor that principally acts on the synthesis of DNA. However, the medical applications are limited due to factors such as short half-life, wide distribution, and various side effects in body. To overcome these limitations, the study on drug delivery systems has been carried out for 5-FU (13). In this study, we used the nanoporous MIL-101-NH_2_-Fe as a carrier for loading the 5-FU as anticancer drug. We expect that the MOF based on iron carboxylate considered in this work with having appropriate surface area and pore size can be a substantial amount of drug loading and release is controlled. For this purpose, experiments verification presence of the drug in the structure in form of quantitative and qualitative was performed. Then, by using the MTT test toxic effects of produced samples on the cell line (MCF7) were investigated. 

## Experimental

*Materials*

Iron (III) chloride hexahydrate (FeCl_3_·6H_2_O), amino terephthalate (NH_2_-H_2_BDC), N,N-dimethyl-formamide (DMF) were purchased from Merck (Darmshtadt Germany), and the 5-fluorouracil was obtained from Sigma (Steinheim, Germany).

*Synthesis of Fe-MIL-101-NH*_2_

The porous MOF Fe-MIL-101-NH_2_ was synthesized by mixing of 0.225 g (1.242 mmol) of NH_2_-H_2_BDC in 7.5 mL of DMF with a solution of 0.675 g (2.497 mmol) of FeCl_3_·6H_2_O in 7.5 mL of DMF. The product was prepared under solvothermal condition in a stainless steel autoclave for 24 h at 110 °C (14). The reaction product was separated by filtration and the crystals washed with DMF and chloroform to remove any residual metal and ligand from product. Then the MOF dried at ambient conditions at 150 °C for 15 h (15). 

*The 5-FU drug loading*

The 5-FU loading in Fe-MIL-101-NH_2_ was carried out by suspension of MOF in drug solution. 120 mg of drug was added to 10 mL of deionized water, and 60 mg Fe-MIL-101-NH_2 _was added to drug solution with weight ratio of 2:1 (drug/MOF). The resulting product was retained under stirring at room temperature for 24 h (13). The drug loaded MOF were centrifuged (14000 rpm) for 10 min at room temperature and washed with deionized water to remove any residual adsorbed drug on the foreign surface. The sample was dried in vacuum conditions at 60 °C. The drug release from MOF was performed by UV-vis spectroscopy at 266 nm to determine the amount of drug loading effecting and entrapment efficiency. The drug calibration curve was also determined at room temperature by mesurment of absorbance.

*Chemical characterization*

FTIR spectra were collected from KBr matrix utilizing a Shimadzuir 460 and used to confirm the drug presence within the MOF matrix. XRD patterns were obtained in a Philips Company X’pert diffractometer with monochromated Cu-Ka radiation. The XRD patterns were used to confirmation of the maintenance of sample crystal structure. The Brunauer–Emmett–Teller (BET) analyses were recorded on Belsorp-mini II Japan, and used to determine the specific surface area, pore size and volume. The TGA analysis was carried out in the temperature range at 20–700 °C under nitrogen gas flow at a heating rate of 10 °C min^–1^. The TGA analysis was determined the chemical composition of samples and the amount of incorporated drug in the MOF matrix. The SEM images were used on (Philips XL 30) with gold coating for structural analysis of samples. Atomic absorption spectroscopy (AAS) was used to measure the iron concentration in the SBF solution. The supernatant SBF solution was discarded, and resuperseded with deionized water to measurement residual iron. 

*In-vitro delivery study*

100 mg of drug-loaded MOF was added into a vial containing 2 mL of SBF with pH 7.4 at 37 °C. At predetermined time intervals, 2 mL of the fresh SBF was replaced and the withdrawn medium was used for determining the drug concentration. The 5-FU concentration was analyzed by UV–vis spectroscopy at 266 nm. The *in-vitro* release study was continued until no 5-FU drug in the withdrawn SBF solution was detected. The 5-FU drug release was performed in duplicate. 

## Results and Discussion

*FTIR spectroscopy*

**Figure 1:**
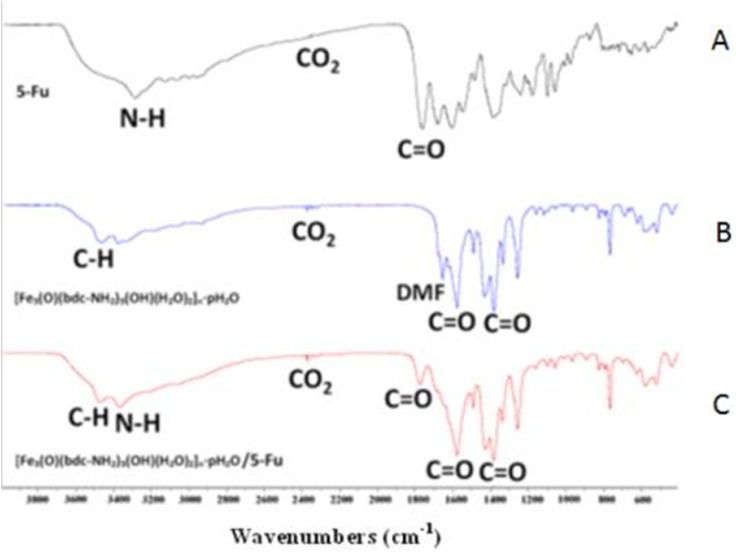
FTIR spectra of pure 5-FU (A), the Fe-MIL-101(NH ) (B), 5-FU loaded Fe-MIL-101(NH ) (C).

MOF and drug FTIR spectra were performed before and after drug loading in the range of 400- 4000 λ^-1^. The FTIR spectrum of Fe-MIL-101(NH_2_) is shown in Figure 1B. The packs of C–H aromatic bands, stretching vibration bands of C-O symmetric and asymmetric of carboxylate in the presence of 2-aminoterfetalat anions, remained DMF molecules in the sample are assigned at 3426, 1435 and 1580, 1620 cm^-1^ respectively. According to literature survey, the peak at 2370 cm^-1^ in samples related to CO_2_, which exist in environment (15). The FTIR spectrum of 5-FU drug is shown in Figure 1A. The N-H amin bonds in 5-FU drug are shown in 3500 cm^-1^. The C=O stretching packs assigned with high intensity at 1390 and 1580 cm^_1 ^in Fe-MIL-101(NH_2_) and 1605 and 1720 cm^-1^ in 5-FU drug. The FTIR spectrum of 5-FU loaded in the MOF is shown in Figure 1C. Based on the results, the FTIR experiment was confirmed qualitatively loaded drug in the sample. Also a small shift of FTIR peaks could be related to the interaction of MOF and drug.

**Figure 2:**
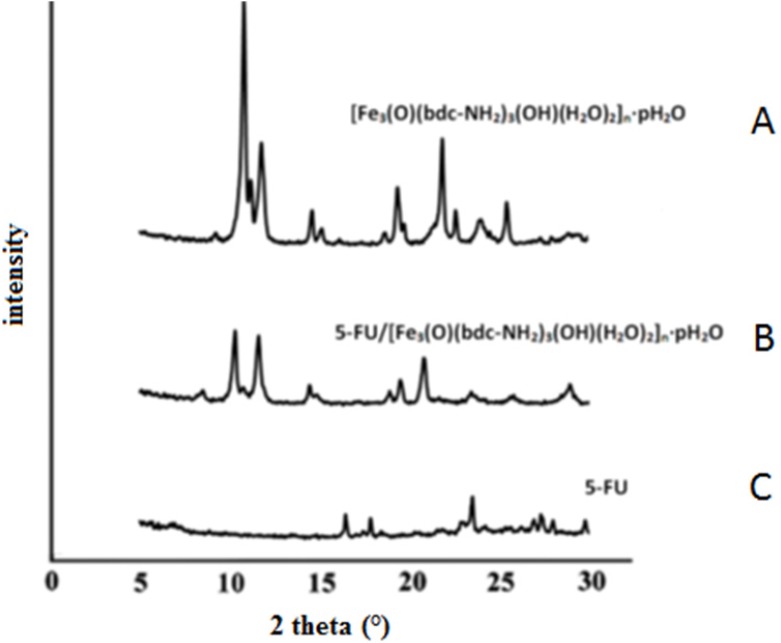
(A) XRD of Fe-MIL-101(NH ), (B) the 5-FU loaded Fe-MIL-101(NH ), (C) 5-FU

**Figure 3:**
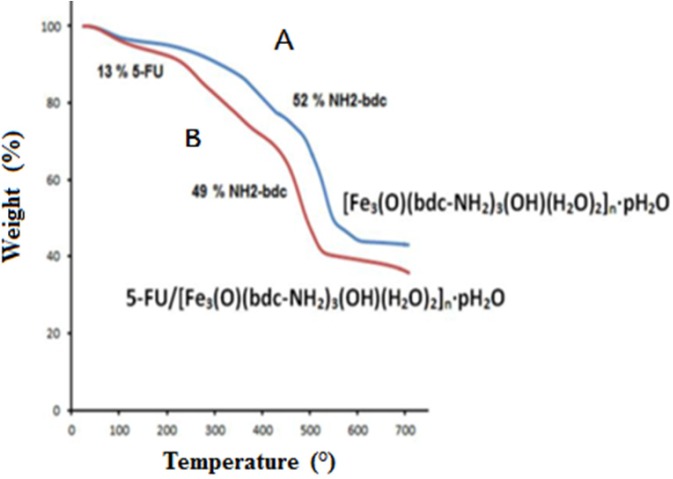
TGA analysis of Fe-MIL-101(NH ) (A) and 5-FU-loaded Fe-MIL-101(NH ) (B)

**Figure 4:**
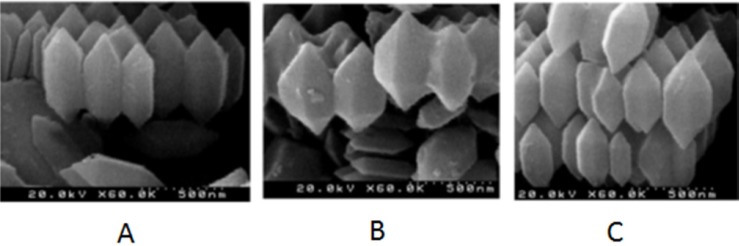
FE-SEM image of 5-FU- Fe-MIL-101(NH ) (A), Fe-MIL-101(NH ) on the SBF solution (B) and Fe-MIL-101(NH ) (C)

**Figure 5:**
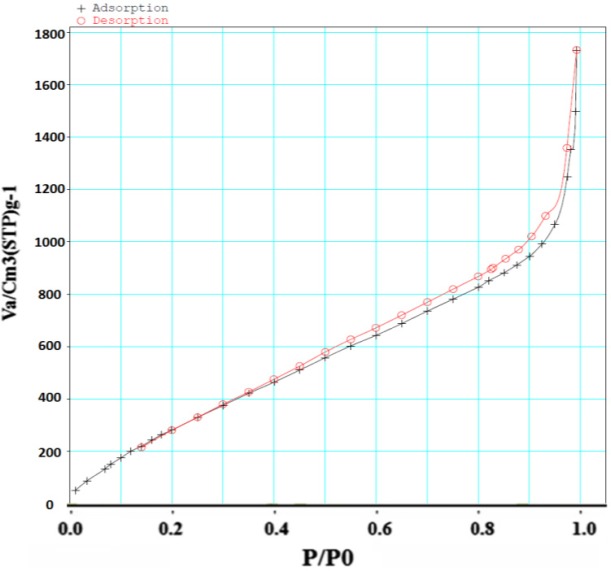
Nitrogen isotherm of Fe-MIL-101(NH )

**Figure 6:**
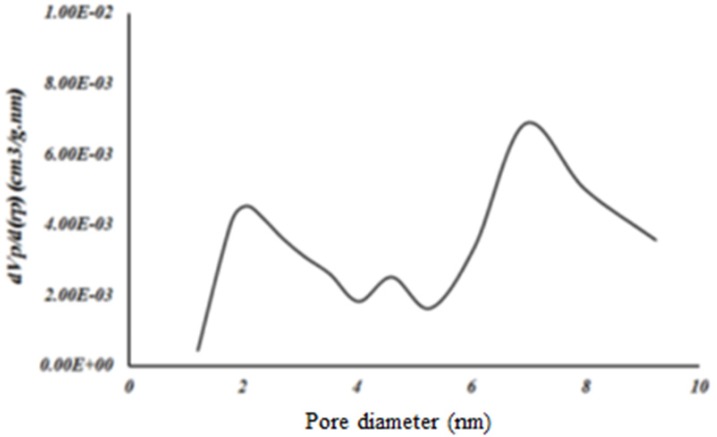
BJH pore size distribution from the adsorption and desorption branch of the isotherm of Fe-MIL-101(NH )

**Figure 7:**
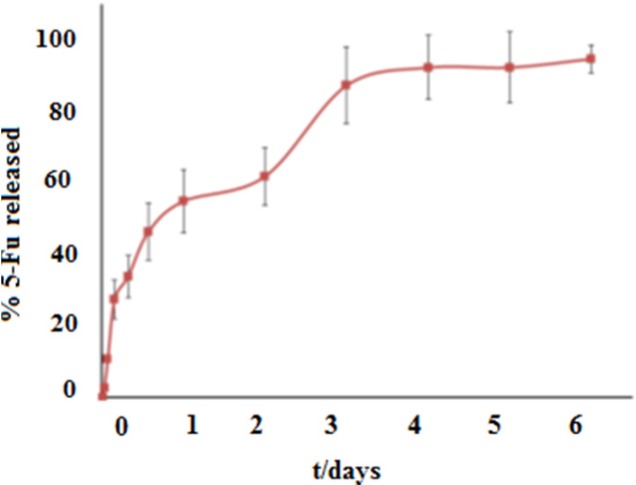
Percentage of the cumulative drug release from 5-FU-loaded Fe-MIL-101(NH ), in SBF pH 7.4 at 37 °C

**Figure 8:**
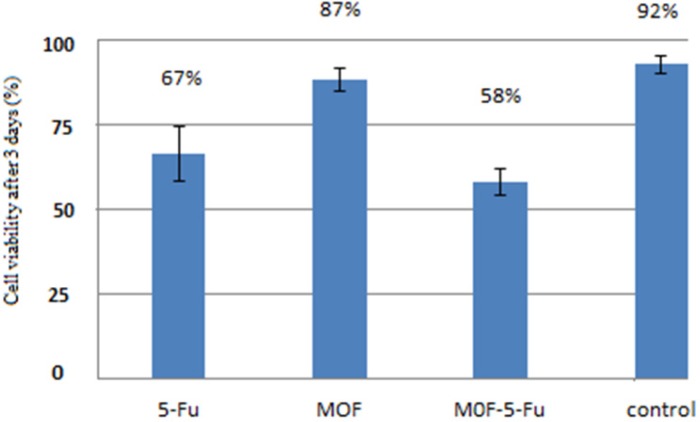
*In-vit* *r* *o* results for three drug delivery systems with (60 μLl/well) concentration againts MCF 7 cell line: 5-FU = the drug 5-Fluorouracil was added directly; MOF = only nanoparticles were added; MOF-5-FU = nanoparticles loaded by 5-Fluorouracil; untreated MCF 7 cells were considered as control. This assay were performed in triplicate(n = 3, mean ± SD).

*XRD diffraction*

The crystal structures of the samples were investigated in the range of 5 to 30° (2θ) by XRD analysis. In Figure 2 the XRD pattern is showed for Fe-MIL-101(NH_2_) (Figure 2 A), the 5-FU loaded Fe-MIL-101(NH_2_) (Figure 2 B) and pure 5-FU drug (Figure 2 C). Based on the results marked peaks of drug do not indicate change in the specific bands of framework after the 5-FU drug loading. Because the drug with the size of 0.5 nm placed within the cavities and channels of porous framework with pore size 2.9 - 3.4 nm. Hence the possibility of taking the drug together within a cavity of MOF limited and also the crystalline property of drug after dissolution in water is decreases partially. In fact the crystalline structure of the MOF was 

maintained after drug loading.

*TGA analysis*

TGA analysis was used by heating samples at a temperature to 700 °C with constant rate of 10 °C /min in an inert atmosphere in the presence of nitrogen gas. Four areas of weight loss were observed for the loaded drug sample. The TGA analysis related to the loss of DMF between 100 and 200 °C (38%), the loss of 5-FU between 200 and 300 °C (13%), and the degradation of the NH2-bdc ligand between 300 to 510 (49%) that this step in TGA analysis corresponded to the decomposition of linkers. According to the results of TGA analysis based on weight loss, the amount of 5-FU drug in the sample was determined about 13% (w/w) (Figure 3). The TGA analysis of MOF and 5-FU loaded MOF are shown in Figure 3 A and B

respectively.

*SEM microscopy*

The SEM results was investigated the morphology and size of MOF and drug-loaded MOF and MOF on the SBF solution. Based on this result the crystal structure of MOF is hexagonal and the MOF has a length of 500 nm and an average diameter of 200 nm (Figure 4). The SEM images of MOF, 5-FU loaded MOF and drug release from 5-FU loaded MOF are shown in Figure 4 C, B, and A respectively. Based on result SEM images the size, crystal structure, and morphology are reminded.

*Nitrogen adsorption*

Surface area of samples was determined by N_2_ adsorption. The Brunauer–Emmett–Teller (BET) analysis is used to evaluate the storage possibility of molecules in the pores and channels of MOF. The holes^, ^diameter and distribution are measured by Barret, Joyner and Halenda (BJH) model. The surface area of MOF was obtained 1486/65 m^2^/g based on BET analysis (Figure 5). The average pore size was measured 2/9-3/4 nm based on BJH analysis that corresponded to the results previously reported for this structure (Figure 6). Accordingly, this MOF with pore sizes larger than 2 nm is placed in categories of meso-porous material (porous materials with pore sizes 2-50 nm) (16) and is appropriated for loading of the small molecule drugs such as 5- FU with an average size of 0.5 nm. 

*Release study*

According to the profile release in Figure 7, 98% of the drug was excluded in the 4 days period from the MOF. In the first hours, the burst drug release was observed, that can be attributed to the drug in its free form and unabsorbed drug within the framework. After the burst release, 5-FU was excluded in two steps from framework. First step of release in the second day is due to the withdrawal of 5-FU drug because of weaker interaction such as van der Waals forces. Second step of release until the fourth day is due to the withdrawal of 5-FU drug because of strong interactions such as hydrogen bonding between drug and ligand. In this study the prolonged 5-FU release from Fe-MIL-101(NH_2_) showed this carrier to be suitable for drug delivery application. According to the only available report, the short delivery time of 48 h for drug 5-FU incorporated in the Cu-BTC MOF (13), the application of Fe-MIL-101(NH_2_) framework as the nano drug delivery carrier is important. 

*Stability test of MOF*

The degradation behavior of MOF was investigated by measuring the iron concentration with AAS at 213.9 nm. Sampling was performed at specific time intervals which were similar to drug discovery study at room temperature. Based on the results, 3% of the total iron was released owing to the MOF degradation that this amount was very small, therefore this MOF is stable in the SBF solution. 

*MTT test*

The MTT test is one of the methods to evaluate the toxicity and this colorimetric method is based on revive and broken yellow crystals tetrazolium to purple formazan in mitochondria of living cells (17, 18). The efficacy of free nanoparticle, drug, and nanoparticle-containing drug were investigated for their ability to eliminate breast cancer MCF-7 cells in 72 h by cell culture experiment. Based on the results in Figure 8, the number of living cancer cells by nanoparticles containing drug is less than the free drug and nanoparticles and the highest cell death (lowest viability) in case of MOF-5-FU was indicated. 

## Conclusion

In this study, the good and prolonged profile release of 5-FU loaded into the Fe-MIL-101-NH_2_ exhibited the potential application of MOF as suitable drug carrier. The 5-FU delivery was monitored for 4 days at 37 °C, pH 7.4 in SBF solution. The presence of drug in the MOF was confirmed by FTIR spectrum and TGA analysis. The 5-FU drug content in samples was determined by TGA analysis to be 13% (W/W). The XRD patterns were showed remained crystalline structure after drug loading in the MOF. By AAS, the stability of MOF was showed stable in aqueous solutions with investigating iron concentration. Also the MTT assay confirmed the performance of the MOF in order to eliminate the cancer cells. The objective of the present study was to develop 5-FU drug delivery application of Fe-MIL-101-NH_2_. In this study we successfully developed a novel carrier for drug loading procedure into the MOF.
